# Pre-competitive anxiety, self-confidence, and technical-tactical performance in professional kickboxing athletes: differences between winners and defeated athletes

**DOI:** 10.3389/fspor.2026.1904878

**Published:** 2026-09-14

**Authors:** Jonatas Deivyson Reis da Silva Duarte, Johan Robalino, Gennaro Apollaro, Cebrail Gençoğlu, Süleyman Ulupınar, Serhat Özbay, Farruh Ahmedov, Carlos Alexandre Fett

**Affiliations:** 1 Postgraduate Program in Health Sciences, Faculty of Medicine, Federal University of Mato Grosso, Cuiabá, Brazil; 2 Faculty of Physical Education, State University of Mato Grosso, Cáceres, Brazil; 3 Centre for Research, Education, Innovation and Intervention in Sport (CIFI2D), Faculty of Sports, University of Porto, Porto, Portugal; 4 Porto Biomechanics Laboratory (LABIOMEP), University of Porto, Porto, Portugal; 5 Department of Neuroscience, Rehabilitation, Ophthalmology, Genetics and Maternal Child Health, University of Genoa, Genoa, Italy; 6 Department of Coaching Education, Faculty of Sport Sciences, Erzurum, Türkiye; 7 Department of Physical Education and Sport, Faculty of Sport Sciences, Erzurum, Türkiye; 8 Department of Physical Education and Sports Teaching, Faculty of Sport Science, Fenerbahçe University, Fenerbahçe, Türkiye; 9 Department of Physical Education and Sports, Samarkand State University, Samarkand, Uzbekistan

**Keywords:** combat sports, K1, martial arts, sport psychology, sport sciences

## Abstract

**Purpose:**

To compare pre-competitive anxiety and self-confidence levels, as well as technical–tactical aspects, between winning and defeated professional kickboxers, and to examine the association of these variables with match outcomes.

**Methods:**

Twenty professional kickboxers (19 males and 1 female) participated in the study, including 10 winners and 10 defeated athletes. Somatic anxiety, cognitive anxiety, and self-confidence were assessed using the Competitive State Anxiety Inventory-2 (CSAI-2). Technical–tactical variables (attacks, efficiency, and efficacy) were analyzed using video recordings of the matches. Data were analyzed using parametric and non-parametric tests, linear mixed models for repeated measures, and binary logistic regression, with effect sizes and reliability indices reported.

**Results:**

There were no significant differences between winners and losers in psychological variables (*p* > 0.05). However, winners demonstrated a higher attack volume (*p* = 0.040; Cohen's *d* = 0.98) and greater efficacy (*p* = 0.024; *d* = 1.10), while no differences were found in efficiency between groups (*p* > 0.05). Additionally, offensive output was descriptively lower in the final round in both groups, although this reduction did not reach statistical significance. Finally, only attack volume showed a trend toward association with victory (*p* = 0.052), corresponding to an approximately 2% increase in the odds of victory (95% CI 1.000 to 1.042).

**Conclusion:**

Competitive success in professional kickboxing is related to greater offensive volume and efficacy, whereas striking efficiency and pre-competitive psychological variables do not differentiate winners from defeated athletes in our sample universe.

## 1 Introduction

Kickboxing is a striking combat sport widely practiced in several countries across different continents (1). It is characterized by a confrontation between two fighters in a ring or on a mat, with the objective of overcoming the opponent through scoring (i.e., valid strikes that hit the target without being blocked by the hands or legs), by knockout (i.e., a state of unconsciousness or inability to continue after a strike), or by technical knockout (i.e., interruption of the fight by the referee, medical team, or corner due to the inability to continue safely) (2, 3). Competitive kickboxing takes place at both amateur and professional levels (4, 5). In the professional context, bouts generally consist of three rounds of three minutes each, with a one-minute rest interval between rounds (6).

**Figure 1:**
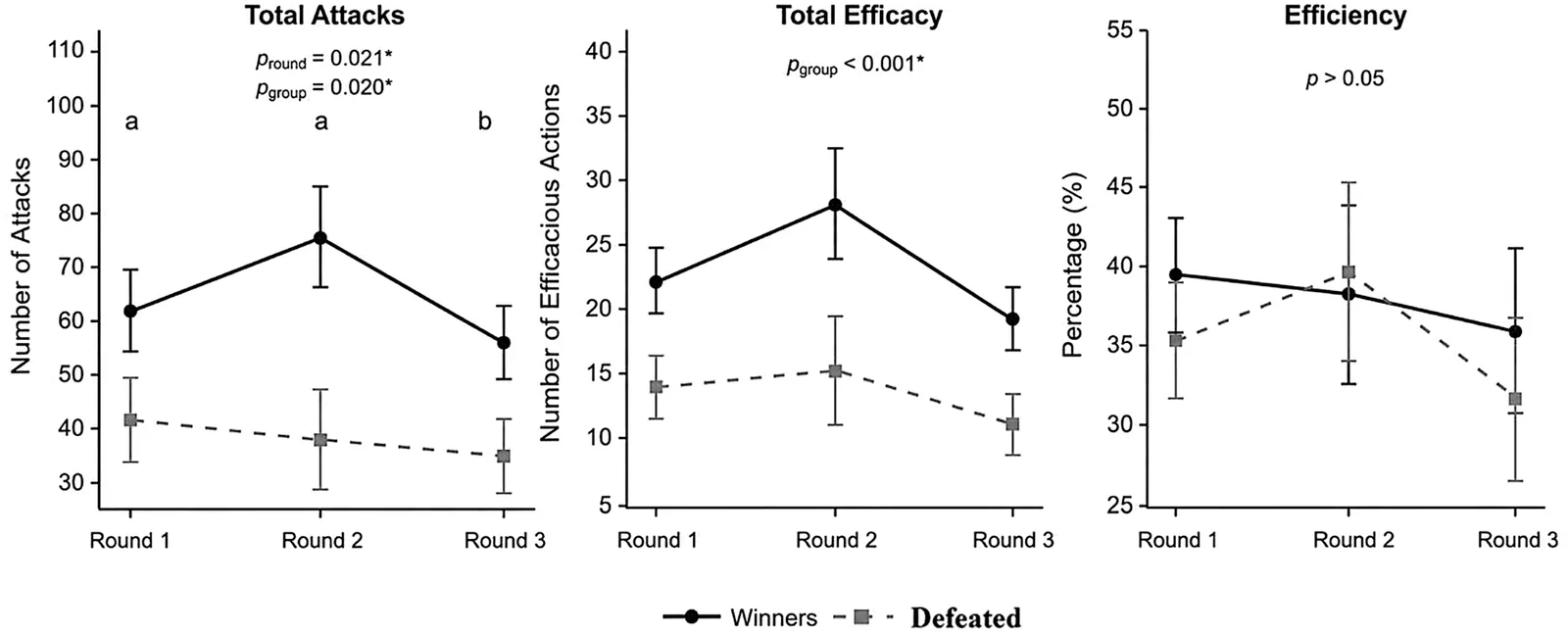
Temporal evolution of technical-tactical performance across three rounds comparing winners and defeated athletes. Data are presented as Estimated Marginal Means (EM Means) ± Standard Error (SE) derived from the Linear Mixed Models, adjusting for cognitive anxiety as a covariate. Solid black lines with circular markers represent Winners, while dashed grey lines with square markers represent defeated. Asterisks (*) indicate significant main effects for the respective factor (*p* < 0.05). The lyrics highlight the differences. No significant main effect of round or round×group interaction was observed for any variable; asterisks denote a significant main effect of group.

Performance in kickboxing is multifactorial and complex, being influenced by different components, including physical factors (7, 8), anthropometric characteristics (9), nutritional aspects (10, 11), chronological age (1, 5), psychological factors (12), and technical-tactical elements (13–16). In this context, sports performance is supported by a multidisciplinary team, composed of sports physicians (17), nutritionists (18), physiotherapists (19), sport psychologists (20), as well as coaches and strength and conditioning coaches (21, 22).

From a psychological perspective, kickboxing bouts impose high demands on cognitive components (12, 23). In this context, pre-competitive anxiety and self-confidence stand out as important psychological dimensions that influence sports performance (24). Anxiety, defined as an emotional state characterized by tension, worry, distress, or fear, may arise significantly in response to real or imagined events and manifests in two main forms: cognitive anxiety and somatic anxiety (25). High levels of anxiety can impair performance by inducing states of agitation, disorientation, and reduced attention, negatively affecting decision-making and technical-tactical execution (26).

Cognitive anxiety involves persistent negative thoughts, worry, and apprehension, which may interfere with attentional focus and decision-making (27). In turn, somatic anxiety is characterized by changes in physiological responses, such as increased heart rate, sweating, and muscle tension, often associated with activation of the hypothalamic–pituitary–adrenal axis, resulting in elevated cortisol levels (28). In contrast, self-confidence refers to an individual's belief in their ability to successfully execute a desired behavior (24).

Technical-tactical aspects also constitute one of the most relevant components of sports performance in kickboxing (14, 29). Technical aspects refer to the strikes permitted by the rules of the modality, such as punches, kicks, and knee strikes (16, 30). In turn, tactical aspects refer to the strategies adopted by coaches and/or athletes with the aim of overcoming the opponent (31). These strategies can be classified as defensive (i.e., counterattacks), offensive (i.e., volume of strikes, combinations, and feints), and mixed (i.e., movements with an offensive and/or defensive nature) (31).

Considering that only one study has analyzed pre-competitive anxiety in simulated kickboxing bouts under laboratory conditions (32), and that the association of technical-tactical aspects has been examined only in relation to physiological markers (i.e., cortisol, lactate, testosterone, among others) (29), no study to date has simultaneously investigated psychological variables and technical-tactical aspects in professional kickboxing, despite psychological variables being an integral part of the complex puzzle of sports performance (24).

Therefore, the aim of the present study was to analyze and compare psychological (i.e., anxiety and self-confidence) and technical-tactical aspects according to match outcomes in professional kickboxing athletes. This study may contribute, from a psychological perspective, to a better understanding of the association between pre-competitive anxiety and self-confidence with the outcomes of professional kickboxing bouts, as well as their relationship with technical-tactical aspects. The main hypotheses were that winning athletes would present lower levels of cognitive and somatic anxiety, as well as higher levels of self-confidence, compared to defeated athletes (33, 34). Additionally, it was hypothesized that winning athletes would exhibit a greater volume of offensive actions, as well as higher efficacy and efficiency of strikes compared to defeated athletes (13–15, 29) and attacks are associated with an increased probability of victory (16).

## 2 Materials and methods

### 2.1 Participants

Although Linear Mixed Models were employed for the primary analyses, an *a priori* sample size estimation was conducted using repeated-measures ANOVA in G*Power software (version 3.1.9.4; Heinrich Heine University Düsseldorf, Düsseldorf, Germany), as it provides a conservative and widely accepted approach for multifactorial repeated-measures designs (14). The following parameters were specified: effect size (*f* = 0.60, large), *α* error probability = 0.05, and statistical power (1−*β* error probability) = 0.80. The analysis included two groups and three repeated measurements (i.e., rounds 1, 2, and 3), assuming a non-sphericity correction (*ε*) of 1.0. Based on these assumptions, the minimum required sample size was 20 participants, corresponding to 8 athletes per group. The selected effect size (*f* = 0.60) was based on the magnitude of the between-round differences in clinch actions reported for official high-level kickboxing championships by Ouergui et al. (14). This assumption was anchored on the technical-tactical outcomes, which constituted the primary repeated-measures variables of the design, and was not appropriate for the psychological variables, for which small-to-moderate effects predominate.

In our study, twenty professional kickboxing athletes participated in the study and were classified as national-level competitors (35). The participants were divided into two groups: winners (*n* = 10) and defeated athletes (*n* = 10). The winners' group consisted of nine men and one woman, whereas the defeated athletes' group comprised exclusively male athletes (*n* = 10). This imbalance reflects the characteristics of the athletes who agreed to participate during the event, despite the inclusion of professional athletes of both sexes in the eligibility criteria. Professional athletes of both sexes were included as an inclusion criterion. Exclusion criteria included incomplete questionnaire responses. All volunteers answered the questionnaire correctly.

### 2.2 Procedures

This cross-sectional study was conducted during a professional kickboxing event held in Cuiabá, Mato Grosso, Brazil. Data collection took place on the day of the competition before the event commenced. Although the event was scheduled to begin at 7:00 p.m. and athletes started arriving at the venue at approximately 6:00 p.m., data were collected individually approximately 60 min before each athlete's bout.

Upon arrival, eligible athletes were approached by the research team and invited to participate in the study. After receiving information about the study procedures, those who agreed to participate provided written informed consent. Subsequently, demographic and sport-related information, including body mass, height, age, and number of professional fights, was collected. Competitive anxiety was assessed using the Competitive State Anxiety Inventory-2 (CSAI-2) (36). Self-reported body mass and height were used, as previous evidence has shown these measures to be valid and strongly correlated with objectively measured values (r > 0.9; *p* < 0.0001) (37).

The study protocol was approved by the Research Ethics Committee involving Human Subjects of the local university and was conducted in accordance with the principles of the Declaration of Helsinki.

### 2.3 Competitive state anxiety inventory-2 (CSAI-2)

To assess pre-competitive anxiety, the CSAI-2 was used. This instrument consists of 17 items distributed across three dimensions: cognitive anxiety (items 2, 5, 8, 11, and 14), somatic anxiety (items 1, 4, 6, 9, 12, 15, and 17), and self-confidence (items 3, 7, 10, 13, and 16) (38). Responses are recorded on a four-point Likert scale ranging from 1 (“not at all”) to 4 (“very much so”). Participants were instructed to respond honestly and accurately. The instrument has demonstrated validity for the Brazilian context (36).

### 2.4 Technical-tactical analysis

The event was fully recorded and broadcast live via YouTube. The recorded bouts were subsequently analyzed by a researcher with five years of experience in kickboxing-related research, who was also a former amateur kickboxing athlete and an experienced referee in the discipline. The technical–tactical analysis included the quantification of the total number of offensive actions performed by each athlete, including punches, kicks, and knees, as well as the number of actions that resulted in scoring. Based on these data, attack efficacy and attack efficiency were calculated using previously described procedures (15, 39). Because attack efficiency is calculated as attack efficacy divided by the total number of attacks, the three technical-tactical variables are not mutually independent.

$$\mathrm{Attack}\;\mathrm{efficacy}=\frac{Total\;points\;scored\;through\;offensive\;actions}{Total\;number\;of\;bouts}$$

$$\mathrm{Attack}\;\mathrm{efficiency}\;(\text{\%})=\frac{Number\;of\;successful\;offensive\;actions}{Total\;number\;of\;offensive\;actions}\;\times \;100\;\;$$

In the context of combat sports, attack efficacy refers to the athlete's ability to convert offensive actions into scoring outcomes, whereas attack efficiency represents the proportion of effective attacks relative to the total number of attacks performed (15). Among the ten bouts analyzed, eight were decided by points, whereas two ended by knockout, one in the first round and the other in the third round. Reliability of the technical–tactical analysis was assessed using the intraclass correlation coefficient (ICC). ICC values for the analyzed variables ranged from 0.993 to 0.998 (*p* < 0.001), indicating excellent reliability. All 10 bouts were analyzed and reanalyzed with an interval of at least one week between assessments. As all ICC values exceeded the commonly accepted threshold of 0.90 for excellent reliability (40), the technical–tactical variables were considered highly reliable.

### 2.5 Statistical analysis

All statistical analyses were conducted using IBM SPSS Statistics (version 26.0; IBM Corp., Armonk, NY, USA). Data normality was assessed using the Shapiro–Wilk test, complemented by visual inspection of Q–Q plots and skewness and kurtosis values (40). Homogeneity of variances was evaluated using Levene's test. The Intra-rater reliability of the notational analysis was quantified using intraclass correlation coefficients [ICC (3,1)] based on a two-way mixed-effects model with absolute agreement. Reliability thresholds were interpreted as poor (<0.50), moderate (0.50–0.74), good (0.75–0.89), and excellent (≥0.90). A subsample of the recorded bouts was reanalyzed with an interval of at least one week between assessments (10 athletes in Round 1 and 8 athletes in Rounds 2 and 3), and ICC values ranged from 0.959 to 1.000.

For cross-sectional comparisons between winners and defeated athletes, independent samples *t*-tests were applied to normally distributed variables, with effect sizes reported as Cohen's *d* and interpreted as trivial (<0.20), small (0.20–0.49), moderate (0.50–0.79), and large (≥0.80) (41). For non-normally distributed variables (i.e., self-confidence), the Mann–Whitney *U* test was used, and effect sizes were calculated using the rank-biserial correlation (*r*). Parametric data were expressed as mean and standard deviation, while non-parametric data were expressed as median and interquartile range [minimum-maximum]. To compare the characteristics between groups, an independent samples t-test was applied. In addition to *p*-values, effect sizes were calculated using Cohen's d to quantify the magnitude of the differences. The effect sizes were interpreted according to standard thresholds (small: 0.2, medium: 0.5, large: 0.8) (41). These analyses were included to provide a more comprehensive interpretation of potential differences between groups. The number of previous professional fights departed from normality in the winners and was therefore compared using the Mann–Whitney U test. Given the restricted sample size, a sensitivity power analysis was additionally conducted in G*Power to determine the minimum effect size detectable with the available sample (*α* = 0.05; 1−*β* = 0.80). Permutation tests with 100,000 resamples were also performed for the between-group comparisons of the technical-tactical variables, as a check that the conclusions did not rest on distributional assumptions.

To examine the temporal evolution of technical–tactical performance across rounds, Linear Mixed Models (LMMs) were implemented. *Round* (1, 2, and 3) and *group* (winner vs. defeated athletes) were included as fixed effects, along with their interaction (*round×group*), and a random intercept for participants was specified to account for within-subject variability. Cognitive anxiety was included as a covariate to mathematically adjust for baseline distress and control for individual psychological variance. Because intra-individual variance in cognitive worry is theoretically established to modulate pacing strategies, decision-making, and physical performance over time (24, 28), this adjustment allowed the LMM to more clearly isolate the pure effect of time (rounds) and group dynamics on technical-tactical deterioration. The covariance structure was selected based on Akaike Information Criterion (AIC). Effect sizes were reported as partial eta squared (*η*ₚ^2^) and interpreted as small (0.01), moderate (0.06), and large (≥0.14) (41), providing an estimate of the proportion of variance explained by each factor. Among the covariance structures examined (unstructured, heterogeneous compound symmetry, first-order autoregressive, compound symmetry and diagonal), the structure minimising the Akaike Information Criterion was retained for each outcome: a first-order autoregressive structure for total attacks and efficiency, and heterogeneous compound symmetry for total efficacy. Model assumptions were verified before interpretation. Normality of the conditional residuals was assessed through histograms, normal Q-Q plots and the Shapiro–Wilk test; homoscedasticity was examined by plotting standardised residuals against predicted values; and influential observations were screened using standardised residuals, with |z| > 3 as the flagging criterion, complemented by leave-one-subject-out refitting. Pairwise comparisons between rounds were adjusted using the Bonferroni correction.

A binary logistic regression model was performed to evaluate the predictive capacity of technical–tactical and psychological variables on match outcome. The dependent variable was match result (0 = defeated, 1 = winner), and predictor variables included total attacks, efficiency, and cognitive anxiety. To preserve model integrity due to the restricted sample size, cognitive anxiety was selected *a priori* over somatic anxiety and self-confidence. This theoretically driven decision was based on the hypothesis that the persistent negative thoughts and apprehension characteristic of cognitive anxiety possess the highest disruptive potential on the rapid visual processing and split-second decision-making paramount in striking combat sports (26, 27), and on the pronounced ceiling effect displayed by self-confidence in the winners, which restricted its variance. Multicollinearity was assessed using variance inflation factors (VIF < 5). Model fit was evaluated using the Hosmer–Lemeshow test, and explanatory power was estimated using Nagelkerke's R^2^. Effect sizes were expressed as odds ratios [Exp(B)] with 95% confidence intervals. Statistical significance was set at *p* < 0.05. Because the Wald statistic is unreliable in small samples, inference for this model was additionally based on the likelihood-ratio test and on Firth's penalized-likelihood regression, both recommended when the number of events per variable is low. Given that the model provides fewer than ten events per predictor, it is reported as exploratory and hypothesis-generating rather than predictive.

## 3 Results

Table 1 presents the sample characteristics (age, height, body mass, and number of professional fights). When comparing winners and defeated athletes, no significant differences were observed in any variable (*p* > 0.05), indicating homogeneity between the groups. The number of previous professional fights was compared using the Mann–Whitney U test (*U* = 61.0, *p* = 0.425).

**Table 1:** Sample characterization.

Characteristics		Winners (*n* = 10)		Defeated (*n* = 10)
		Mean ± SD [95% CI]		Mean ± SD [95% CI]
Age (years)		25.6 ± 5.4 [21.8–29.4]		29.1 ± 7.1 [24.0–34.2]
Height (meters)		1.76 ± 0.07 [1.71–1.81]		1.79 ± 0.06 [1.74–1.83]
Weight (kg)		77.4 ± 10.2 [70.1–84.7]		78.7 ± 9.4 [72.0–85.4]
Professional fights (*n*)		10.8 ± 14.6 [0.4–21.2]		5.5 ± 4.4 [2.3–8.7]

SD, standard deviation; kg, kilogram; *n*, number of fights; CI, confidence interval.

The psychological dimensions (i.e., somatic anxiety, cognitive anxiety, and self-confidence) did not show significant differences between the winner and defeated athletes' groups (*p* > 0.05) (Table 2). Effect sizes and their confidence intervals are reported in Table 2. The largest psychological effect was observed for self-confidence (rank-biserial *r* = 0.33), followed by cognitive anxiety (Hedges' *g* = 0.67, 95% CI −0.15 to 1.85), whereas somatic anxiety showed a trivial effect (*g* = −0.05, 95% CI −0.99 to 0.82). The sensitivity analysis indicated that the design was powered to detect only very large between-group differences (*d* ≥ 1.32).

**Table 2:** Pre-competitive anxiety state between winners and defeated.

Variables	Winners (*n* = 10)		Defeated (*n* = 10)
	Mean ± SD [95% CI]		Mean ± SD [95% CI]
Somatic Anxiety	1.74 ± 0.51 [1.38–2.10]		1.77 ± 0.58 [1.35–2.19]
Cognitive Anxiety	1.80 ± 0.31 [1.58–2.02]		1.54 ± 0.42 [1.24–1.84]
Self-confidence^a^	4.0 (3.0–4.0) [3.56–4.04]		3.5 (2.6–4.0) [3.06–3.86]

SD, standard deviation; CI, confidence interval.

a expressed as median and interquartile range (minimum and maximum).

Winners demonstrated significantly greater total attacks (*p* = 0.041, Hedges' *g* = 0.95, 95% CI 0.06 to 1.92) and total efficacy (*p* = 0.024, g = 1.06, 95% CI 0.16 to 2.05) compared to defeated athletes, both representing large effect sizes, indicating substantial practical differences between groups. However, no significant differences in efficiency were found between the groups (*p* > 0.05) (Table 3). Permutation tests confirmed both differences (total attacks *p* = 0.043; total efficacy *p* = 0.025).

**Table 3:** Technical–tactical performance between winners and defeated.

Technical–Tactical	Winners (*n* = 10)		Defeated (*n* = 10)
	Mean ± SD [95% CI]		Mean ± SD [95% CI]
Total Attacks	178.15 ± 87.30 [115.70–240.60]*		108.35 ± 48.72 [73.50–143.20]
Total Efficacy	64.95 ± 28.26 [44.74–85.16]*		38.70 ± 18.14 [25.72–51.68]
Efficiency	38.92 ± 10.88 [31.14–46.70]		36.75 ± 11.03 [28.86–44.64]

SD, standard deviation; CI, confidence interval.

* Significantly higher compared to defeated athletes (*p* < 0.05).

Linear mixed-effects models (LMMs), using the Satterthwaite approximation for degrees of freedom, were applied to examine performance across rounds. The main effect of round on total attacks was not statistically significant (*F* = 2.041, *p* = 0.145, *η*ₚ^2^ = 0.11). Mean offensive output was descriptively lower in the third round (45.4 actions) than in the first (51.8) or the second (56.7), but none of the pairwise comparisons remained significant after Bonferroni correction (all *p* ≥ 0.18). A significant main effect of group was observed (*F* = 7.647, *p* = 0.013, *η*ₚ^2^ = 0.30), reflecting a consistently higher level of offensive activity in winners compared to defeated athletes throughout the bout. The round×group interaction was not statistically significant (*F* = 2.395, *p* = 0.106, *η*ₚ^2^ = 0.12) (Figure 1).

For total efficacy, a significant main effect of group was observed (*F* = 9.084, *p* = 0.008, *η*ₚ^2^ = 0.36), with no significant effect of round (*p* = 0.090) or interaction (*p* = 0.638). No significant effects were found for efficiency (*p* > 0.05). Additionally, the psychological covariate (cognitive anxiety) did not exert a significant influence on performance outcomes within the models (*p* > 0.05).

The logistic regression model (Table 4) identified total attacks the variable showing the strongest tendency toward association with match outcome**.** The Wald test did not reach conventional significance (*B* = 0.020, *SE* = 0.011, *p* = 0.052), whereas the likelihood-ratio test (*p* = 0.016) and Firth's penalized-likelihood estimation (*p* = 0.037) did. The corresponding odds ratio [Exp(B) = 1.021; 95% CI 1.000 to 1.042] indicates that each additional offensive action was associated with an approximately 2% increase in the odds of victory. Because the Wald-based confidence interval includes the null value, this estimate is reported as an exploratory association.

**Table 4:** Binary logistic regression model for predicting match outcome.

Predictor	*B*	S.E.	*p*-value	Exp(B)	95% CI for Exp(B)
Total Attacks	0.020	0.011	0.052	1.021	[1.000, 1.042]
Cognitive Anxiety	2.239	1.728	0.195	9.384	[0.318, 277.313]
Efficiency	0.069	0.059	0.244	1.071	[0.954, 1.203]

B, unstandardized regression coefficient; SE, standard error; Exp(B), odds ratio; CI, confidence interval.

Cognitive anxiety and efficiency did not emerge as significant predictors (*p* > 0.05), and their associated effect estimates suggest limited explanatory contribution within the multivariate model.

## 4 Discussion

This study aimed to compare levels of anxiety, self-confidence, and technical-tactical aspects between winning and defeated professional kickboxers, as well as to analyze the possible associations between these variables and match outcomes. Overall, the results partially supported the established hypotheses. No significant differences were observed in somatic anxiety, cognitive anxiety, or self-confidence between winners and defeated athletes, contrary to the initial expectations. On the other hand, the number of attacks and the efficacy of strikes were higher in winning athletes, in line with the study hypotheses. Additionally, a tendency was observed for the number of attacks to act as a predictor of victory. In this sense, the findings of the present study suggest that, at the professional level, technical-tactical variables appear to have greater discriminative power than pre-competitive psychological variables.

In combat sports, evidence suggests a consistent association between elevated levels of anxiety and competitive performance. In boxers and jiu-jitsu athletes, cognitive anxiety was higher in defeated athletes (42, 43). Similarly, in kurash (a combat sport), higher levels of anxiety were also observed in defeated athletes (44). In a classic study on taekwondo, self-confidence was found to be higher in winners, whereas cognitive and somatic anxiety were higher in defeated athletes (34), a pattern similar to that observed in karate (33). Additionally, in simulated kickboxing bouts, defeated athletes also presented higher levels of anxiety (45).

Unlike our findings and the other studies discussed here, winning international-level judokas exhibited higher levels of cognitive anxiety (46). This discrepancy may be related to differences in competitive level and sport-specific demands. Athletes competing at the international level are often exposed to greater performance expectations and competitive pressure, which may increase pre-competitive anxiety (47). However, elite athletes may also possess superior coping skills, allowing them to maintain or even enhance performance despite experiencing elevated anxiety levels (48). Therefore, the impact of anxiety on performance may depend not only on its intensity but also on how athletes interpret and manage these symptoms (49).

Despite the convergence of these findings with the results of the present study, caution is warranted when making direct comparisons. This is because these investigations were conducted with amateur athletes (33, 34, 42–44, 46), whereas the kickboxing study (45) was carried out in a simulated context under controlled conditions. In this sense, this study appears to be among the first to examine these variables in professional kickboxing during an official competition, thus providing a novel contribution to the literature. Nevertheless, further studies are needed to confirm these findings across different modalities and competitive levels.

Several factors may explain the absence of differences in pre-competitive anxiety levels between the groups. More experienced athletes, for example, tend to exhibit better control of emotional responses, resulting in more stable levels of pre-competitive anxiety (42). In the present study, although participants did not present a high number of competitions at the professional level, it is important to consider that athletes in this category generally accumulate prior experience at the amateur level, participating in numerous bouts throughout their sporting careers (50). This accumulated experience may contribute to the development of emotional self-regulation strategies, attenuating potential differences between winners and defeated athletes.

In this context, it is also important to highlight that anxiety does not necessarily exert an exclusively negative effect on sports performance (26). According to classical theoretical models, such as that proposed by Weinberg and Gould (24), the relationship between physiological activation (somatic anxiety) and performance follows an inverted “U” pattern, in which moderate levels of anxiety may facilitate performance, whereas very low or excessively high levels tend to impair it. In this sense, it is possible that the athletes assessed presented anxiety levels within an optimal range, which could explain the absence of significant differences between the groups.

Additionally, athletes with better physical and technical-tactical preparation tend to present higher levels of self-confidence and lower levels of anxiety (51). In this regard, it is important to note that professional athletes are generally exposed to higher training loads, both in duration and weekly frequency, compared to amateur athletes (50), which may also contribute to better control of psychological responses in competitive contexts.

In the present study, winning athletes performed a greater number of attacks and demonstrated higher efficacy compared to defeated athletes (representing the only variable that discriminated between winners and defeated athletes). Similar results have been observed in amateur kickboxing, where winners of world championships executed a higher volume of attacks (14). Consistently, evidence in professional kickboxing also indicates that winning athletes are more effective and deliver more strikes (13). These findings are in agreement with the results of the present study.

Similar patterns of offensive technical-tactical behavior have also been identified in other striking combat sports, such as boxing (52), taekwondo (53), and karate (54). Taken together, this evidence suggests that the predominance of offensive strategies, combined with greater efficacy of actions, constitutes a determining factor for competitive success in striking combat sports. A recent study demonstrated that defensive actions using the hands and legs reduce the chances of victory by 4.2% to 10.4% (16).

The absence of differences in strike efficiency between winners and defeated athletes, together with the higher number of scoring actions achieved by winners, may be explained by a greater ability to chain combinations of strikes, often ending with scoring attacks using the upper and/or lower limbs. In this context, physical conditioning plays a key role in maintaining effective technical-tactical performance throughout the rounds (39). Offensive actions are primarily supported by anaerobic energy systems (55, 56).

In the present study, offensive output was descriptively lower in the final round, although this reduction did not reach statistical significance**.** Similar findings have been reported in investigations involving kickboxing sparring, in which high-intensity actions also decreased progressively. That decline has been attributed to accumulated physical fatigue. Given the limited sample size, the present study was likely underpowered to detect an effect of this magnitude, and the question therefore remains open for adequately powered designs.

The progressive reduction in offensive actions across rounds may also be interpreted in relation to mental fatigue (57). Although the present study did not directly assess mental fatigue, combat sports require continuous vigilance, rapid response selection, inhibition of inappropriate actions, and tactical adjustment under pressure (58, 59). Recent evidence indicates that cognitive fatigue can impair vigilance, explosive performance, neuromuscular output, and asymmetry-related performance indicators (58–60). Therefore, future studies should simultaneously assess mental fatigue, technical-tactical performance, and physiological responses to determine whether reductions in offensive volume are associated with physical fatigue alone or with a combination of physical and cognitive factors in kickboxing.

Additionally, a trend was observed for the number of attacks to act as a predictor of victory. While the unit-level odds ratio indicates an approximately 2% increase in the odds of winning per single additional attack, its practical relevance is best understood through meaningful combat increments. For instance, executing 10 or 50 additional attacks over the course of a bout would correspond to odds ratios of approximately 1.23 (95% CI 1.00 to 1.51) and 2.78 (95% CI 0.99 to 7.77), respectively. Because these intervals include the null value, these findings represent preliminary associations. In this sense, the findings of the present study suggest that, **within the present sample,** at the professional level, technical-tactical variables may have greater discriminative power than pre-competitive psychological variables. Another study that analyzed the discriminative capacity of physiological variables (i.e., heart rate, testosterone, lactate, among others) and technical-tactical variables between winning and defeated kickboxers in a simulated bout found differences only in the technical-tactical aspects (29). These findings are consistent with the present results, in which no differences were observed in psychological variables, just as no differences were found in physiological variables in the study by Ouergui et al. (29).

### 4.1 Limitations

However, some limitations should be considered in the present study. Although the sample size was sufficient to identify significant differences in technical-tactical aspects, it does not allow for generalization of the results. Another important aspect is that the effect size adopted for the sample size calculation was large (f = 0.60), which may not have been sufficient to detect significant differences in psychological variables with small or moderate effect sizes. The reduced sample is related to the difficulty of recruiting professional athletes as volunteers, a limitation frequently reported in the literature (61). Furthermore, the majority of combat sports practitioners are amateur athletes, with professionals being less represented in scientific studies (50). In this regard, investigations involving professional kickboxers are particularly relevant for advancing the understanding of this phenomenon. A sensitivity analysis indicated that the design was powered to detect only very large between-group differences (*d* ≥ 1.32). This constraint was asymmetric across outcome domains: detecting the observed technical-tactical effects would have required approximately 14 to 18 athletes per group, whereas the psychological effects would have required 31 to 34. The absence of significant differences in the psychological variables should therefore not be read as evidence of absence but as a plausible Type II error. Similarly, with ten events and three predictors the logistic model provides fewer than ten events per variable, and its estimates should be regarded as exploratory.

Another limitation is the marked sex imbalance in the sample, as only one female athlete participated and was allocated to the winners' group. Consequently, potential sex-related differences could not be examined or statistically controlled. Although the athlete met all eligibility criteria and was retained to preserve the ecological validity of the sample, the findings should primarily be interpreted as reflecting male professional kickboxing athletes. Future studies should recruit a larger and more balanced sample of female athletes to determine whether these findings are consistent across sexes.

Additionally, although the use of questionnaires is widely employed in sport psychology and supported by evidence of validity (36), the possibility of response biases cannot be ruled out, such as social desirability and subjective interpretation of the items (62). Furthermore, it is important to note that data collection conducted immediately prior to competition could reflect distinct cognitive states and potentially be more sensitive to the competitive context. However, this procedure was not feasible due to the need to preserve athletes' concentration and the potential perception of impaired performance. Finally, the fact that not all athletes volunteered may imply a selection bias and the sex distribution, with only one woman in one group, limit the generalizability of the findings and preclude sex-based analyses.

## 5 Conclusion

Competitive success in professional kickboxing appears to be related to a higher volume of offensive actions and greater efficacy. In contrast, strike efficiency did not differ between winners and defeated athletes, suggesting that match outcomes may be more closely related to the ability to produce and sustain offensive actions than to the relative accuracy of these actions. Additionally, no significant differences were observed in the pre-competitive psychological variables between winners and defeated athletes in the present sample. However, this finding should be interpreted with caution given the limited sample size and the possibility of a Type II error.

## 6 Practical applications

An offensive tactical approach may represent a valid strategy for kickboxing athletes; however, its effectiveness likely depends on technical accuracy, defensive skills, and the ability to tolerate fatigue. Coaches should also consider that a higher attack frequency may increase exposure to counterattacks. Based on the present findings, training strategies aimed at improving athletes' ability to sustain offensive actions throughout the bout may be considered. In this context, approaches designed to enhance fatigue tolerance, such as high-intensity interval training, strength-endurance training targeting the primary muscle groups involved in combat, and sport-specific sparring, may represent useful options within a comprehensive training program.

From a psychological perspective, although pre-competitive variables did not differentiate winners and defeated athletes, psychological skills training may still play a relevant role in maintaining performance under competitive pressure. Accordingly, strategies such as arousal regulation, attentional focus, mentalization, mindfulness practices, and emotional control may be considered to help athletes sustain offensive output and the quality of decision-making throughout the bout.

## Data Availability

The raw data supporting the conclusions of this article will be made available by the authors, without undue reservation. Requests should be directed to the corresponding author.
